# Photoelectric Properties of Planar and Mesoporous Structured Perovskite Solar Cells

**DOI:** 10.3390/ma15124300

**Published:** 2022-06-17

**Authors:** Steponas Ašmontas, Aurimas Čerškus, Jonas Gradauskas, Asta Grigucevičienė, Remigijus Juškėnas, Konstantinas Leinartas, Andžej Lučun, Kazimieras Petrauskas, Algirdas Selskis, Laurynas Staišiūnas, Algirdas Sužiedėlis, Aldis Šilėnas, Edmundas Širmulis

**Affiliations:** Center for Physical Sciences and Technology, Savanorių Ave. 231, LT-02300 Vilnius, Lithuania; aurimas.cerskus@ftmc.lt (A.Č.); jonas.gradauskas@ftmc.lt (J.G.); asta.griguceviciene@ftmc.lt (A.G.); remigijus.juskenas@ftmc.lt (R.J.); konstantinas.leinartas@ftmc.lt (K.L.); andzej.lucun@ftmc.lt (A.L.); kazimieras.petrauskas@ftmc.lt (K.P.); algirdas.selskis@ftmc.lt (A.S.); laurynas.staisiunas@ftmc.lt (L.S.); algirdas.suziedelis@ftmc.lt (A.S.); aldis.silenas@ftmc.lt (A.Š.); edmundas.sirmulis@ftmc.lt (E.Š.)

**Keywords:** advanced energy materials, perovskite, solar cell, thin film, power conversion efficiency

## Abstract

The high efficiency of perovskite solar cells strongly depends on the quality of perovskite films and carrier extraction layers. Here, we present the results of an investigation of the photoelectric properties of solar cells based on perovskite films grown on compact and mesoporous titanium dioxide layers. Kinetics of charge carrier transport and their extraction in triple-cation perovskite solar cells were studied by using transient photovoltage and time-resolved photoluminescence decay measurements. X-ray diffraction analysis revealed that the crystallinity of the perovskite films grown on mesoporous titanium dioxide is better compared to the films grown on compact TiO_2_. Mesoporous structured perovskite solar cells are found to have higher power conversion efficiency mainly due to enlarged perovskite/mesoporous -TiO_2_ interfacial area and better crystallinity of their perovskite films.

## 1. Introduction

It has become increasingly urgent to look for renewable energy sources and improve the existing ones due to the ever-increasing energy demand accompanied by the exhaustion of organic fuels, global warming, and the current political situation. One of the most promising and environmentally friendly energy sources is electricity generated by solar cells. The organic–inorganic hybrid perovskite-based solar cells (PSC) show a promising future for photovoltaic technology [1]. During the last decade, the power conversion efficiency (PCE) of PSCs has grown from 3.8% to more than 25.7% [2], making them a very rapidly advancing technology and a popular topic in the field of solar cells. The high performance of PSCs can primarily be attributed to their excellent optoelectronic properties, such as (1) strong light absorption through the entire visible spectrum range, enabling the use of thin perovskite films [3,4]; (2) high carrier mobility [5,6]; (3) long carrier diffusion length [7,8]; (4) high defect tolerance [9]; and (5) long lifetime of the generated charge carriers [10,11]. To realize the good performance of a PSC, high-quality perovskite film containing a low density of defects must be employed in a single device [12]. The defects in a perovskite layer may affect both charge carrier transport and their extraction processes [13], and they can induce trap states, causing increased charge carrier recombination rate and, thereby, the reduced power conversion efficiency of a PSC [14]. To obtain perfectly operating perovskite devices, carrier transport and extraction layers are also important in addition to high-quality perovskite films [15,16]. There are several basic requirements for the transport layers: (1) good energy-level alignment with a perovskite for efficient charge transfer, (2) high mobility of charge carriers to guarantee their fast transport, (3) high transmittance to reduce the optical energy loss, (4) good stability, and (5) easy processing and low cost [16]. Two typical PSC structures are widely used: one is a planar heterojunction architecture [7,17], and another is a mesoporous structure [18,19]. Planar perovskite solar cells are advantageous because they have simple and scalable configurations [20]. However, the highest PCE is achieved with mesoscopic PSC, in which a mesoporous metal oxide layer plays an important role as the charge transport channel, scaffold for loading the light-absorbing materials, and electron–hole separator [21].

Titanium dioxide (TiO_2_) has been mostly used as an electron transport layer (ETL) in PSCs due to its wide bandgap, E_g_ = 3.2 eV, suitable for minimized parasitic absorption, an appropriate conduction band alignment with a perovskite for efficient electron injection, and good electron-transport capability [22]. The quality of ETL plays an important role in improving the performance of PSCs [23]. An ETL transports photoinduced electrons away from the perovskite, and at the same time, it serves as a blocking layer preventing direct contact between the holes and fluorine-doped tin oxide (FTO) [24,25]. Therefore, TiO_2_ layers should be uniform, pinhole-free, and should completely cover the surface of FTO [26]. The optimal thickness of crystalline TiO_2_ depends on the deposition technique and post-treatment of the layers. In the case of planar PSCs, the thickness of an ETL is in the range of 20–30 nm [26,27,28]. The deposition of a mesoporous (mp-TiO_2_) layer on crystalline titanium dioxide provides a reduced carrier transport length and increased interfacial contact area, thereby facilitating efficient charge carrier collection and yielding the highest reported PCE values [29]. It has been shown that the structural properties of the mp-TiO_2_ layer, such as particle size, thickness, and porosity, have a significant influence not only on the performance of PSCs but also on the layer’s photoelectric properties [13,26,30,31,32]. The transient photovoltage (TPV) measurements showed that the TPV decay strongly depends on the porosity of TiO_2_ film, and there is a significant correlation between the TPV decay characteristics and the performance of the perovskite solar cells [13]. It was established that the time-resolved photoluminescence (TRPL) decay also strongly depends on the size of TiO_2_ nanoparticles [30], the porosity of the TiO_2_ film, mesoscopic structure, and perovskite morphology formed therein [13]. TRPL spectroscopy is a powerful technique to study excited charge carrier recombination in perovskite layers that notably determine the PCE of PSCs [14].

In this paper, we report experimental results of the kinetics of the charge carrier transport and their extraction obtained by performing transient photovoltage and time-resolved photoluminescence decay measurements with the intention to elucidate the correlation between the electronic processes in perovskite layers and the performance of perovskite solar cells.

## 2. Fabrication of Perovskite Films and Their Characterization

**Figure 1:**
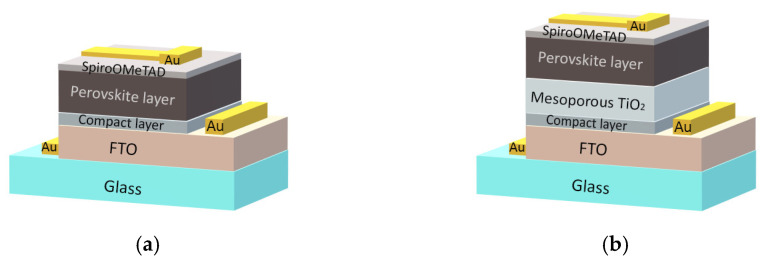
Schematic configuration of the cells fabricated for the study: (**a**) without porous TiO_2_ layer, (**b**) with porous TiO_2_ layer. The compact TiO_2_ layers with and without Nb_2_O_5_ were formed using the ALD and spray pyrolysis methods, respectively.

In this study, two types of perovskite solar cells, with and without a porous TiO_2_ layer, were fabricated. Their schematic configurations are shown in Figure 1. The cells also differ in the formation and composition of the compact TiO_2_ layer: in one case, the compact TiO_2_ layers were formed by the conventional spray pyrolysis method; in another case, the compact TiO_2_ layers containing niobium oxide (Ti_1–x_Nb_x_O_2−_mixed titanium niobium oxide) were deposited using the Atomic Layer Deposition (ALD) method. In both cases, the 25 × 25 mm^2^ glasses coated with a transparent fluorine-doped tin oxide (FTO) layer (TEC 10, Ossila, UK) were used as substrates. The composition of precursors, their concentrations and purity grades, the sequence of procedures and equipment, and the parameters of the layers (ETL, perovskite layer, and hole transport layer), all used for the fabrication of the perovskite cells, are detailed in our previous papers [33,34].

Thin, compact Ti_0.93_Nb_0.07_O_2_ layers were deposited in a “Fiji F200” ALD reactor (Cambridge Nano Tech, Cambridge, MA, USA) using similar procedures as described in [35]. Precursors for titanium and niobium oxides were tetrakis dimethylamino titanium (TDMAT, 99.9%; STREM Chemicals Inc., Newburyport, MA, USA) and niobium ethoxide (Nb(OEt)_5_, 99.9%; STREM Chemicals Inc., Newburyport, MA, USA), and deionized water was used as an oxygen source. The mixed oxide was formed by depositing a single layer of niobium oxide for every 20 layers of deposited titanium oxide. The deposition consisted of 420 monolayers and resulted in an approximately 30 nm-thick coating.

We should note that porous TiO_2_, perovskite, and hole transport layers were formed over the ALD-mixed titanium niobium oxide layer using the same precursors and procedures as in the case of compact TiO_2_ layers formed by spray pyrolysis. Cs-containing Cs_x_(MA_0.17_FA_0.83_)_1–x_Pb(I_0.83_Br_0.17_)_3_ perovskite layers of the same composition with x = 0.1 were grown for both cell types. The 70 nm-thick Au contacts were deposited in the vacuum chamber of the thermal evaporation equipment “VAKSIS PVD Vapor-5S_Th” (Vaksis R&D and Engineering, Ankara, Turkey) on the top of the hole transport layer (Spiro-OMeTAD from Sigma-Aldrich, St. Louis, MO, USA, was used for its formation) and on the open side of FTO (see Figure 1).

The surface morphology and cross-section of the films were examined using a scanning electron microscope (SEM) (Helios NanoLab 650, FEI, Hillsboro, OR, USA).

X-ray diffraction (XRD) patterns were obtained using an X-ray diffractometer (XRD) (SmartLab, Rigaku, Tokyo, Japan) equipped with a 9 kW power rotating Cu anode X-ray source and theta/theta goniometer. The patterns were measured with Bragg–Brentano geometry in the 2Θ range of 10–65°.

**Figure 2:**
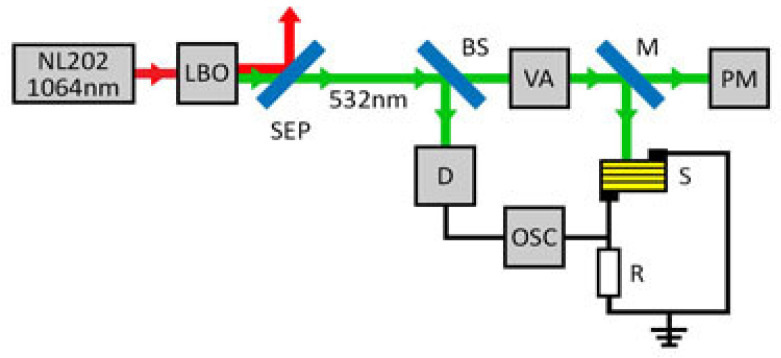
The scheme of the experimental setup of transient photovoltage measurement. NL202 is the diode-pumped Nd:YAG laser; LBO is the nonlinear crystal for the second harmonic generation; SEP is the Nd:YAG laser harmonic separator; BS is the beam splitter plate; VA is the variable attenuator for 532 nm laser beam; M is the removable high-reflectance mirror; S is the sample solar cell; R is the load resistor; PM is the optical power meter; D is the optical signal reference detector; OSC is the digital storage oscilloscope.

The transient photovoltage of the solar cells was measured using the setup shown in Figure 2. A diode-pumped, frequency-doubled Nd:YAG-LBO laser NL202 (Ekspla Ltd., Vilnius, Lithuania) generated 7 ns-long pulses of 532 nm radiation. To prevent the sample from heating, a low pulse repetition rate of 5 Hz was used. A harmonic separator (dichroic beamsplitter; Eksma Ltd., Vilnius, Lithuania) was employed to reflect the rest of the infrared beam and transmit the 532 nm radiation. A part of the laser beam was directed to an optical signal reference detector (Standa Ltd., Vilnius, Lithuania) using a beam splitter plate. A variable attenuator (Eksma Ltd., Vilnius, Lithuania) consisting of a Brewster-type polarizer and quartz half wave-plate allowed changing the power of the incident laser beam. The average power of the laser radiation was measured by an optical power meter PM400 (Thorlabs Inc., Newton, NJ, USA). The maximum power density incident on the sample was about 0.5 mWcm^−2^. A laser beam was directed onto the sample S with a removable high-reflectance mirror (M). The transient photovoltage and the laser pulse were recorded using a digital storage oscilloscope DSO6102A (Agilent Technologies Inc., Santa Clara, CA, USA), and the laser pulse shape was registered by the high-speed detector 11HSP-FS1 (Standa Ltd., Vilnius, Lithuania).

The light from a continuous-wave (CW) Ar-ion laser or pulsed 532 nm microchip laser (Standa Ltd., Vilnius, Lithuania) was used for excitation in a standard photoluminescence (PL) measurement setup with a 1 m monochromator and thermoelectrically cooled photomultiplier tube working in the photon counting regime. More details of this technique are described in our previous paper [36]. The power of the lasers was varied with a linear variable metallic neutral density filter. The samples were held in a vacuumed optical cryostat during all CWPL and time-resolved photoluminescence (TRPL) measurements at room temperature.

The photovoltaic characteristics of the perovskite solar cells were measured using Keithley 2602A (Keithley Instruments Inc., Cleveland, OH, USA) equipment. The 100 mW/cm^2^ irradiance was produced by an AM 1.5 spectral lamp (Newport model 67005, Newport Corp., Irvine, CA, USA) placed at an appropriate distance.

## 3. Results and Discussion

**Figure 3:**
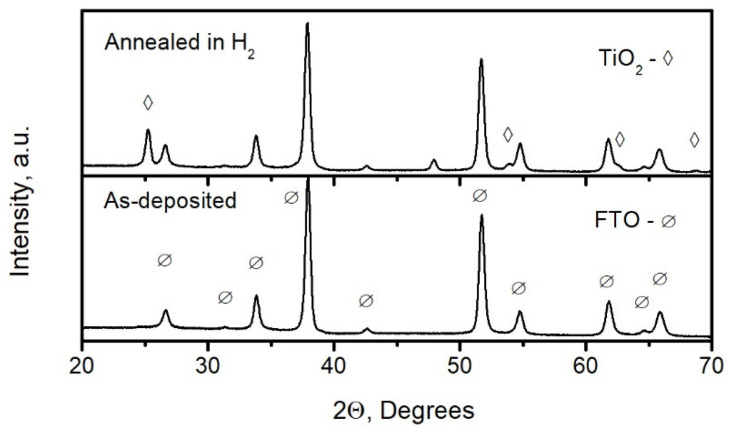
XRD patterns of the Ti_0.93_Nb_0.07_O_2_ film before (as-deposited) and after annealing in a H_2_ atmosphere.

Figure 3 presents the XRD patterns of the Ti_0.93_Nb_0.07_O_2_ film before (as-deposited) and after annealing in an H_2_ atmosphere at 400 °C for 50 min. The ramping-up rate was 5 °C/min, and the ramping-down rate was 3 °C per minute. It can be seen that the XRD pattern of the as-deposited film presents only FTO peaks. The XRD peaks of TiO_2_ (anatase) emerged on the XRD pattern of the Ti_0.93_Nb_0.07_O_2_ film annealed in a H_2_ atmosphere. The electrical resistivity of the Ti_0.93_Nb_0.07_O_2_ film decreased from 2 × 10^2^ to 3.5 × 10^−3^ Ω cm during the annealing.

**Figure 4:**
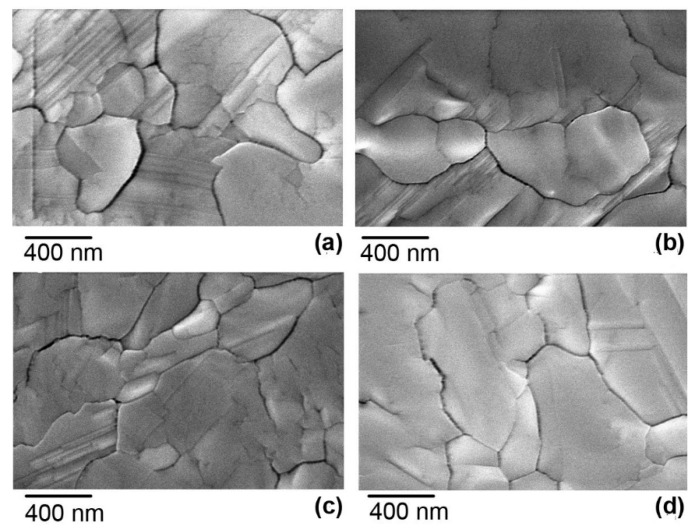
Surface SEM images of perovskite films grown on different types of titanium dioxide layers: (**a**) Film A on the c-TiO_2_ layer, (**b**) Film B on the mp-TiO_2_ layer/c-TiO_2_, (**c**) Film C on the compact ALD-TiO_2_ layer, and (**d**) Film D on the mp-TiO_2_ layer/ALD-TiO_2_.

The top-view SEM images of the triple cation perovskite films grown on titanium dioxide using different methods are shown in Figure 4. The first perovskite (Film A) was grown on a compact titanium dioxide (c-TiO_2_) layer formed by the conventional spray pyrolysis method (FTO/c-TiO_2_/perovskite). The second perovskite film (Film B) was grown on the mesoporous TiO_2_ layer (FTO/c-TiO_2_/mp-TiO_2_/perovskite). The third perovskite film (Film C) was grown on the compact TiO_2_ layer deposited using the ALD method (FTO/ALD-TiO_2_/perovskite). The fourth perovskite film (Film D) was grown on the mesoporous TiO_2_ layer (FTO/ALD-TiO_2_/mp-TiO_2_/perovskite). It can be seen that the morphology of the perovskite films and grain sizes are similar and the surfaces are flat and smooth, in agreement with other reports elsewhere [23,32].

**Figure 5:**
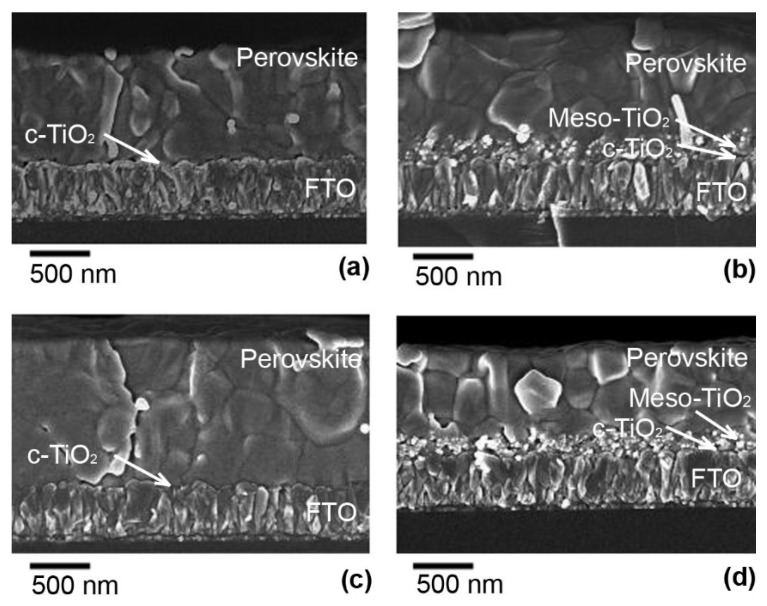
Cross-sectional SEM images of perovskite films on different TiO_2_ layers: (**a**) Film A on the compact TiO_2_ layer, (**b**) Film B on the mp-TiO_2_ layer/c-TiO_2_, (**c**), Film C on the compact ALD-TiO_2_ layer, and (**d**) Film D on the mp-TiO_2_ layer/ALD-TiO_2_.

The cross-sectional SEM images of the same perovskite films are shown in Figure 5. It is seen that the morphology and grain distribution in the films grown on the compact TiO_2_ are similar (Figure 5a,c). The thickness of Films A and C was (880 ± 30) nm and (1200 ± 50) nm, respectively. However, the interface between Film A and c-TiO_2_ formed by spray pyrolysis (Figure 5a) contained some small cavities. The perovskite material grown on mesoporous TiO_2_ has penetrated between the nanocrystals of the mp-TiO_2_ layer (Figure 5b,d). The thickness of Films B and D was (880 ± 50) nm and (720 ± 50) nm, respectively. Though the surface images of the perovskite films (Figure 4) show grains with dimensions greater than 1000 nm, the cross-section images show that the films were composed of stacked grains but not of ones spanning over the whole thickness of the layer (Figure 5b,d).

**Figure 6:**
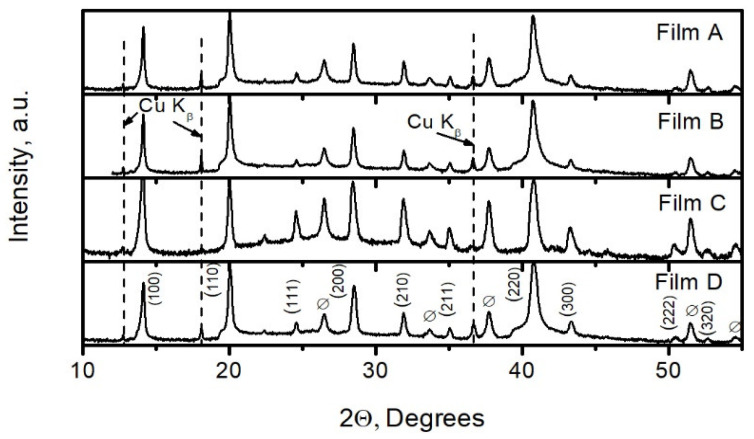
XRD patterns of perovskite films on different titanium dioxide layers: Film A is on the compact TiO_2_ layer, Film B is on the mp-TiO_2_ layer/c, Film C is on the compact ALD-TiO_2_ layer, and Film D is on the ALD-TiO_2_/mp-TiO_2_ layer.

The XRD patterns of the perovskite films grown on different TiO_2_ layers are depicted in Figure 6. Most of the XRD peaks corresponded to 2Θ values of the cubic FA_5/6_Ma_1/6_PbBr_0.5_I_2.5_ phase (ICDD PDF card #01-085-6374). The rest of the peaks were attributable to FTO. Visually, the XRD patterns of the films were very similar. However, in the case of Film C, the perovskite XRD peak 100 was of higher intensity than peak 110, in contrast to the other patterns. This could mean that the crystallites of the Film C have a prevailing orientation in the [100] direction while the rest of the films have another prevailing orientation, namely, [110].

The XRD peaks of the perovskite films formed on TiO_2_ by spray pyrolysis seem to be sharper. This means that the crystallinity of the perovskite films grown on mesoporous titanium dioxide is better compared to the films grown on compact TiO_2._ The values of full width at half-maximum (FWHM) of the perovskite peaks 100, 110, and 220 are presented in Table 1. The size of a crystallite, D, and the value of micro-deformation, Δd/d (d is the interplanar spacing), both calculated using the Hall method, are also presented in the table. The FWHM values of Films A and B were smaller in comparison to those formed using the ALD method (Films C and D). Respectively, the size of crystallites was larger, and microdeformation was smaller in Films A and B, thus pointing to lower dislocation density in these films.

**Figure 7:**
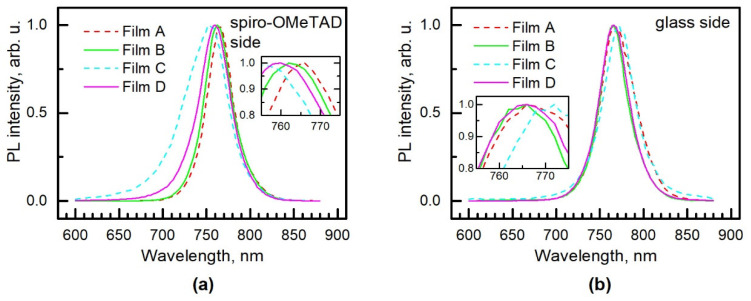
CWPL spectra of PSC measured from (**a**) Spiro-OMeTAD side and (**b**) Glass side. Dashed and solid lines indicate planar and mesoporous structured cells, respectively. The insets show the corresponding enlarged part of peak position.

Figure 7 shows CW photoluminescence spectra measured from both sides of the planar and mesoporous structured solar cells. For most of the samples, the peak position of the top side (spiro-OMeTAD side) spectrum is within the ±3 nm range centered at 762 nm. This finding agrees well with the other results of triple cation perovskites containing 10% of Cs [34,37]. However, the 10 nm blue shift is detected in the case of the Film C sample. Nevertheless, the peak position of the bottom side (glass side) is less scattered among the samples (Figure 7b), but it is slightly redshifted compared to the spiro-OMeTAD side case. Analyzing the “same side” PL results, one can observe that the peak position of the samples containing the mp-TiO_2_ layer has lower energy values than those of the samples with the c-TiO_2_ layer; but, again, except for the sample with Film C from the spiro-OMeTAD side. A study of the integrated PL intensity *I*_PL_ dependence on laser power density *P* in the 1 mW/cm^2^ to 65 mW/cm^2^ range revealed that the Film C sample has the weakest dependence: its slope coefficient *k* ($\log I_{\mathrm{PL}}\propto k\log P$) equals 0.95 when measured from the spiro-OMeTAD side. The coefficient *k* values are presented in Table 2. As a rule, the slope is below 1 for the free-to-bound and donor–acceptor transitions, and stays between 1 and 2 for the free-exciton or bound-exciton transitions [38]. The magnitude of *k* depends on the power density *P* and on material properties such as radiative and competitive nonradiative recombination probabilities [39] or the contribution of deep defects [40]. All these anomalies of Film C could be related to the smaller size of its crystallites. Therefore, a blue shift is observed in the CWPL spectrum from the spiro-OMeTAD side, and it changes the recombination probabilities, leading to a lower *k* value. Additionally, the decrease in the *k* value could result from donor–acceptor or free-bound transitions, including the contribution of the deep defects.

**Figure 8:**
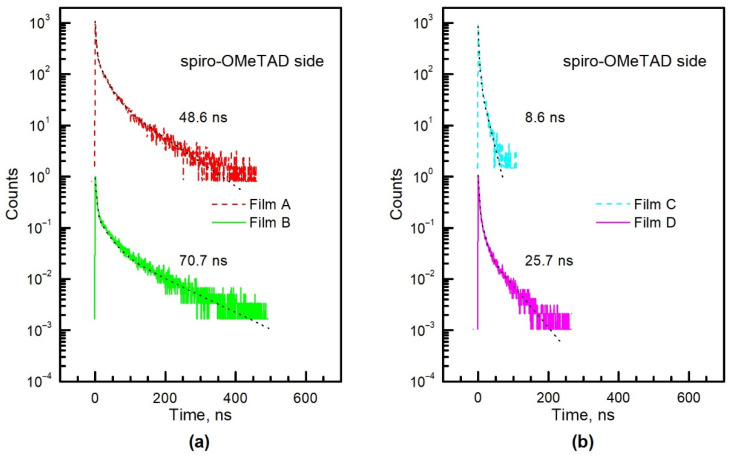

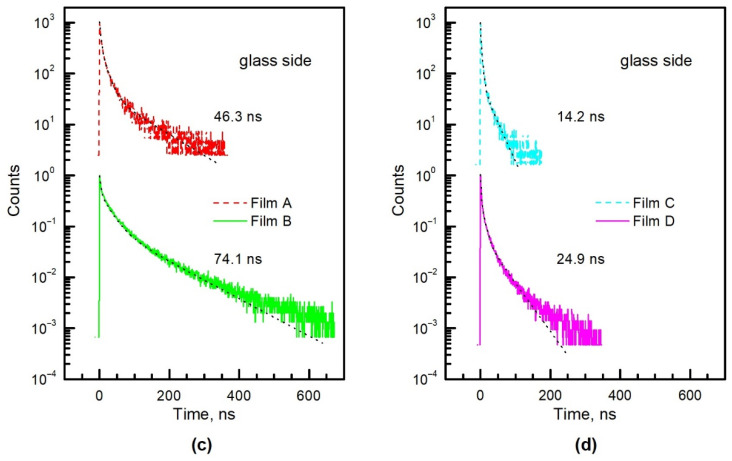
PL decay transients of PSC measured from the spiro-OMeTAD side (**a**,**b**) and from the glass side (**c**,**d**). Black dotted lines indicate triple exponent approximation fitting, and the numbers (in ns) are the averaged decay time constants.

Time-resolved photoluminescence decays measured both from spiro-OMeTAD and glass sides under a pulsed laser fluence of 0.1 μJ/cm^2^ are displayed in Figure 8. It shows the results of the longest average decay times obtained at different positions of all 20 samples (five samples from each series were tested). The transients were fitted using triple exponential approximation [33,41,42],

(1) $$I_{\mathrm{PL}}\left(t\right)=\overset{3}{\underset{i\;=\;1}{\sum }}A_{i}e^{-\frac{t}{\tau_{i}}}$$

where fitting parameters *A_i_* and *τ_i_* are the amplitude and the decay time constant of the *i*th term. The fitting results according to Equation (1) are displayed in Figure 8 as a dotted line. The fitting parameters and the calculated average decay time,

(2) $$\tau_{\mathrm{dec}}=\sum_{i\;=\;1}^{3}A_{i}\tau_{i}^{2}/\sum_{i\;=\;1}^{3}A_{i}\tau_{i},$$

are presented in Table 2. One can conclude that longer decay times are typical of perovskites on mesoporous TiO_2_ (samples Film B and D lead against Film A and C, respectively) and of layers formed using the conventional spray pyrolysis method (samples Film A and B lead against Film C and D, respectively). Meanwhile, Film C demonstrates the shortest decay times that can be influenced by the carrier surface recombination, which correlates with the results of structure analysis. The triple exponential fitting model is not good enough in some cases. Thus, we should additionally consider the processes of trapping and de-trapping [14].

**Figure 9:**
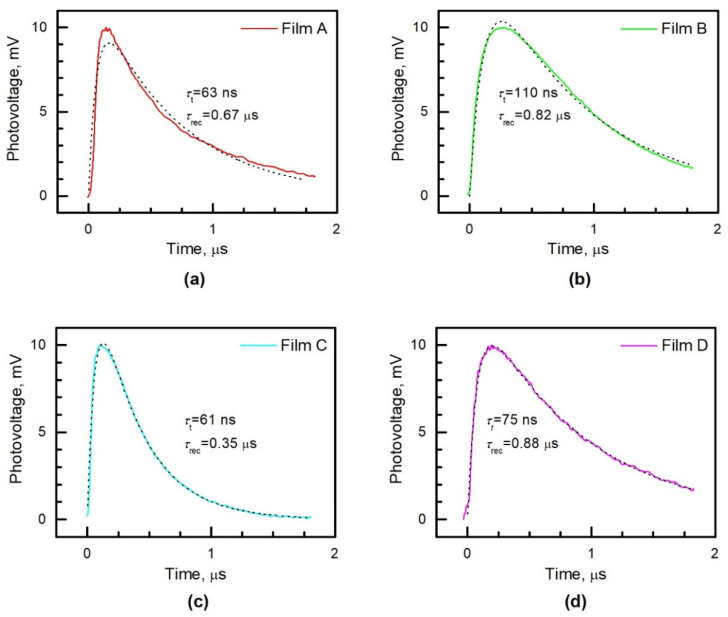
Photovoltage decay transients of the solar cells fabricated on the base of different perovskite films: (**a**) Film A, (**b**) Film B, (**c**) Film C, (**d**) Film D. Black dotted lines indicate fitting according to Equation (3), and *τ_t_* and *τ*_rec_ are the electron transport and recombination time constants, respectively.

Photovoltage decay transients of the solar cells fabricated on different perovskite films are depicted in Figure 9. The photovoltage reaches its maximum value with a delay of approximately 100 to 200 ns after laser excitation. The dependence of photovoltage on time can be described by the following equation [13]:

(3) $$V\left(t\right)=V_{0}\left(\frac{e^{-t\tau_{t}}-e^{-t\tau_{\mathrm{rec}}}}{1\tau_{\mathrm{rec}}-1\tau_{t}}\right)$$

where *τ_t_* is the time constant of electron transport inside the perovskite layer, *τ*_rec_ is the electron recombination time, and *V*_0_ is the initial photovoltage. Fitting results calculated according to Equation (3) are depicted in Figure 9 as a dotted line. The redistribution of excited charge carriers inside the perovskite layer is determined mainly by their diffusion since the electric field is weak here. Therefore, *τ_t_* is approximately equal to *L*^2^/*D*, where *L* is the layer thickness and *D* is the bipolar diffusion coefficient. The estimated *D* values are 0.12, 0.07, 0.25, and 0.07 cm^2^/s for perovskite samples Film A, B, C, and D, respectively. These values of bipolar diffusion coefficient are typical of spin-coated perovskite layers [5,7,43,44,45]. It should be noted that the diffusion coefficient is higher in planar than in mesoporous structured perovskite solar cells, as was observed earlier in [44], whereas the lifetime of generated charge carriers is longer in mesoporous structured perovskite solar cells due to the better crystallinity of mesoporous structured perovskite films.

**Figure 10:**
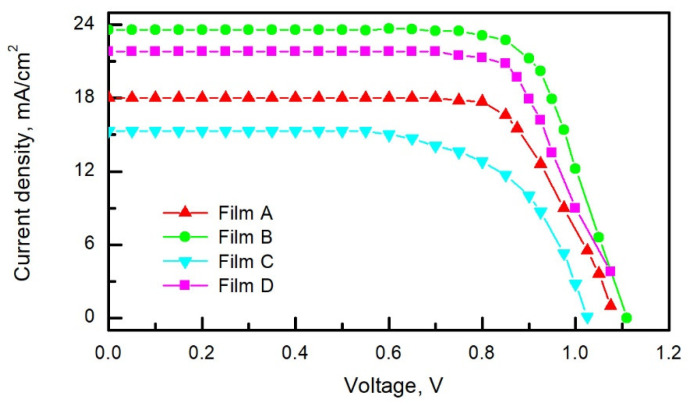
Current–voltage characteristics of the solar cells fabricated on different perovskite films under 100 mW/cm^2^ spectral lamp irradiance.

**Figure 11:**
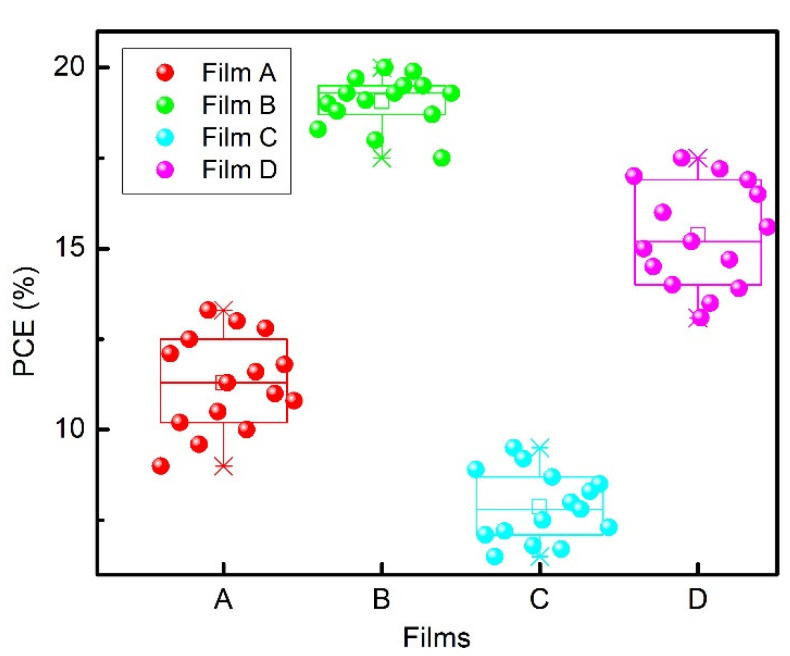
Statistical distribution of the power-conversion efficiency of perovskite solar cells based on different perovskite films.

The current versus forward bias voltage characteristics of the solar cells fabricated on different perovskite films are presented in Figure 10. It can be seen that the mesoporous structured PSCs show better performance than the planar ones. The values of photovoltaic quantities of the best PSCs are presented in Table 3, and statistics of the power-conversion efficiency of 60 perovskite solar cells fabricated on different perovskite films are depicted in Figure 11. The high PCE of mesoporous structured perovskite solar cells is mainly determined by a high value of the short circuit current resulting from an enlarged perovskite/mp-TiO_2_ interfacial area compared to the planar PSC case.

It is worth noting that the high crystallinity of mesoporous structured perovskite also has a positive effect on the performance of PSCs because uniform perovskite films with high surface coverage and minimum defects are beneficial for the transportation of both electrons and holes [46]. Additionally, time-resolved photoluminescence decay and transient photovoltage measurements showed that the lifetime of generated charge carriers in mesoporous structured perovskite solar cells is longer than that in planar PSCs, and as a consequence, the performance of the mesoporous structured PSCs is better. Negligible hysteresis of the current–voltage characteristics of the perovskite Cs_0.1_(MA_0.17_FA_0.83_)_0.9_Pb(I_0.83_Br_0.17_)_3_ solar cells is also a noteworthy fact, which was observed earlier in other works [33,37,47]. The planar PSCs fabricated on the base of Films A and C show the lowest PCE values, and this finding can be related to the shorter lifetime of charge carriers in these films resulting from their lower crystallinity (see Figure 8 and Figure 9). On the other hand, the mesoporous structured perovskite solar cells fabricated on the base of Film B demonstrate the best PCE due to the high crystallinity of the perovskite film, giving a reason for the long lifetime of the generated charge carriers. In addition, the crystallites of Film B are the largest (see Table 1). This also greatly contributes to the improved performance of the mesoporous structured perovskite solar cells [47]. Thus, we see a good correlation between electronic processes in perovskite layers and the performance of perovskite solar cells.

## 4. Conclusions

An experimental study of the photoelectric properties of planar and mesoporous structured perovskite solar cells was conducted. It was established that the crystallinity of perovskite films grown on mesoporous titanium dioxide is better than that of films grown on a compact TiO_2_. Transient photovoltage and time-resolved photoluminescence decay measurements show that the lifetime of charge carriers is also longer in mesoporous structured PSCs. The presented experimental data reveal the relationship between photovoltage decay characteristics and PSC performance, as both of them are affected by the mesoscopic structure of the layered structure. It was found that the mesoporous structured perovskite solar cells have a higher PCE than the planar ones, mainly due to an enlarged perovskite/mp-TiO_2_ interfacial area and longer lifetime of generated charge carriers. These results provide a basis for improved further material growth and device fabrication to achieve better performance of perovskite solar cells.

## Figures and Tables

**Table 1:** The FWHM values of perovskite XRD peaks 100, 110, and 220, crystallite size D, and the value of microdeformation Δd/d in different perovskite films.

Sample	FWHM_(100)_	FWHM_(110)_	FWHM_(220)_	D, nm	Δd/d
Film A	0.071	0.091	0.167	181.0 ± 30.3	0.25 ± 0.12
Film B	0.065	0.083	0.164	189.0 ± 82.3	0.23 ± 0.05
Film C	0.131	0.197	0.338	73.7 ± 15.0	0.29 ± 0.04
Film D	0.105	0.134	0.243	100.0 ± 12.3	0.30 ± 0.17

**Table 2:** Results of PL spectra analysis. *k* is the slope of log *I*_PL_ versus log *P* curve; *A_i_* and *τ_i_* are the amplitude and the decay time constant of the *i*th term in Equation (1); *τ*_dec_ is average decay time calculated using Equation (2).

	Spiro-OMeTAD Side				Glass Side			
	Film A	Film B	Film C	Film D	Film A	Film B	Film C	Film D
*k*	1.37	1.57	0.95	1.22	1.36	1.42	1.11	1.49
A_1_	0.85	0.82	0.40	0.78	0.64	0.49	0.50	0.69
*τ*_1_, ns	2.5	2.9	0.79	2.2	3.2	2.7	0.96	1.4
A_2_	0.19	0.16	0.37	0.23	0.37	0.38	0.44	0.29
*τ*_2_, ns	23.1	27.4	3.3	10.4	15.6	27.7	4.7	9.5
A_3_	0.05	0.05	0.08	0.05	0.04	0.11	0.07	0.07
*τ*_3_, ns	95.9	124	15.8	54.4	105	119	28.2	45.9
*τ*_dec_, ns	48.6	70.7	8.6	25.7	46.3	74.1	14.2	24.9

**Table 3:** Photovoltaic parameters of the solar cells on the base of different perovskite films. *V*_oc_ is the open-circuit voltage; *J*_sc_ is the short circuit current; FF is the fill factor; PCE is the power-conversion efficiency.

Film	*V*_oc_, V	*J*_sc_, mA·cm^−2^	FF, %	PCE, %
Film A	1.09	18	72	14.1
Film B	1.11	23.5	77	20.0
Film C	1.03	15.1	63	9.8
Film D	1.1	21.8	73	17.5

## Data Availability

No new data were created or analyzed in this study. Data sharing is not applicable to this article.
